# Basal Cell Carcinoma: An Old Friend with Multiple Faces

**DOI:** 10.3390/cancers17060993

**Published:** 2025-03-15

**Authors:** Maria Chiara Sergi, Francesca Ambrogio, Mario Della Mura, Joana Sorino, Gerardo Cazzato

**Affiliations:** 1 Unità Operativa Complessa di Oncologia Medica, Ospedale “Mons. A.R. Dimiccoli” Asl BT, Viale Ippocrate, 15, 70051 Barletta, Italy; 2 Section of Dermatology and Venereology, Department of Precision and Regenerative Medicine and Ionian Area (DiMePRe-J), University of Bari “Aldo Moro”, 70124 Bari, Italy; francesca.ambrogio@policlinico.ba.it; 3 Section of Molecular Pathology, Department of Precision and Regenerative Medicine and Ionian Area (DiMePRe-J), University of Bari “Aldo Moro”, 70124 Bari, Italy; mariodellamura1@gmail.com (M.D.M.); j.sorino@studenti.uniba.it (J.S.); gerycazzato@hotmail.it (G.C.)

**Keywords:** basal cell carcinoma, metastasis, Hedgehog pathway inhibitors, immunotherapy, chemotherapy

## Abstract

BCC is the most prevalent form of skin cancer, characterized by diverse clinical and pathological subtypes ranging from indolent to highly aggressive forms. While the majority of BCC cases are treated effectively with surgery or local therapies, locally advanced BCC (laBCC) and metastatic BCC (mBCC) pose significant therapeutic challenges. Recent advances in HHIs and immunotherapy have transformed the treatment landscape in such cases. However, resistance and intolerance to these treatments necessitate alternative approaches, including chemotherapy. Platinum-based agents such as cisplatin and carboplatin have shown limited efficacy but remain viable options in rapidly progressive cases. Among the therapeutic innovations to be explored, further lines of immunotherapy as well as combination therapies involving immunotherapy and targeted therapy have been proposed. This review synthesizes the current understanding about BCC subtypes, risk stratification, and emerging treatments, with a particular focus on laBCC and mBCC.

## Simple Summary

The most common type of skin cancer is basal cell carcinoma (BCC), which has a variety of clinical and pathological subtypes that range from mild to highly aggressive. The treatment landscape has changed due to recent developments in immunotherapy and Hedgehog pathway inhibitors (HHIs), but alternative strategies are emerging in resistant patients. Additionally, radiation therapy is also taken into consideration. The present knowledge of BCC subtypes, risk assessment, and new treatment approaches is summarized in this review.

## 1. Introduction

BCC represents the most common type of skin cancer globally, predominantly affecting areas of chronic sun exposure in older individuals [1,2,3,4]. Its indolent nature and low metastatic potential generally enable effective management with surgical excision or localized therapies [1,2,3,4,5,6]. However, a subset of BCCs, characterized by high-risk pathological features or advanced disease stages, challenges conventional treatment paradigms [5,6]. Over the past decade, the development of targeted therapies such as HHIs and immune checkpoint inhibitors (ICIs) has dramatically improved outcomes for patients with laBCC and mBCC [5,6]. Despite these advancements, clinical scenarios involving resistance, intolerance, or limited accessibility to these therapies highlight the need for alternative systemic approaches. Among these, chemotherapy, though traditionally considered of limited utility, has demonstrated anecdotal efficacy in specific cases, offering a critical option in aggressive and rapidly progressing disease [1,2].

This review explores the evolving landscape of BCC treatment, with a focus on clinical and histological subtypes, emerging biomarkers, and the role of novel systemic therapies. Furthermore, we aim to provide an updated framework for clinicians navigating complex therapeutic decisions in the management of advanced BCC.

## 2. Clinical Features of BCC

BCC is the most common form of skin cancer and typically presents as a slow-growing, locally invasive tumor. Clinically, BCCs are generally well circumscribed and often appear on sun-exposed areas of the body, such as the face, neck, ears, and upper torso in older adults [1,2,3,4,5,6,7].

The European standardized rate increased significantly for both sexes between 1973 and 2009, quadrupling from 40 to 165 per 100,000 person-years for men and from 34 to 157 for women. The rate increased even more from 2002 to 2009, reaching an EAPC of 7.9% (95% CI, 6.2–9.7) for women and 6.8% (95% CI, 5.3–8.3) for men [8].

The distinction between low-risk and high-risk BCCs, which is based on both clinical and pathological criteria, is crucial for their clinical management.

According to NCCN Guidelines of Basal Cell Skin Cancer, three different risk categories can be identified: **low-risk BCC**, **high-risk BCC** and **advanced BCC** [9,10]. The high-risk features consist in: location on head, neck, hands, feet, pretibial, and anogenital area (any size); location on trunk or extremities with a tumor size ≥ 2 cm; poorly defined clinical borders; recurrent BCC; immune suppressed patient; history of prior radiotherapy; high-risk pathological features (*vide infra*). When anyone of the high-risk features listed above is noted, the lesion is categorized as high-risk BCC, while in all other cases, it can be classified as low-risk BCC. The third category, represented by advanced BCC, includes both laBCC and mBCC. For each one of these categories, different treatment options are warranted [9,10].

Likewise, the European Association of Dermato-Oncology (EADO) formulated a staging system offering a more detailed approach that goes beyond the simple low and high-risk division, introducing a more detailed classification. It consists of four stages, reflecting different clinical situations and distinguishing between ‘easy-to-treat’ BCC and ‘difficult-to-treat’ BCC [1,9,10].

**EADO-stage I** includes easy-to-treat, low-risk BCCs. More than 90% of BCCs belong to this category, being easily controlled through standard surgery or a range of alternative blind treatments during the initial months or years after diagnosis.

**EADO-stage II** is divided into IIA, representing common BCCs that are somewhat difficult to treat due to factors like location or prior recurrences, and IIB, that includes BCCs that are difficult to treat primarily because of a high number of BCCs.

**EADO-stage III** covers large or destructive BCCs, with subcategories based on their location or involvement of critical areas, thus correlating with the difficulty of surgical asportation.

Finally, **EADO-stage IV** is for very rare cases with distant metastasis [1,9,11].

In this way, the EADO classification provides a more nuanced and practical approach in everyday clinical practice. It helps clinicians make more informed decisions for complex cases, considering not just the risk of metastasis but also the difficulty of treatment, the potentiality to cause significant local damage and the specific features of each case [1,10,11].

In synthesis, low-risk BCCs represent the majority of cases and are typically smaller well-defined tumors, located in non-critical areas of the body and have a low likelihood of recurrence. Their management is generally straightforward, often involving local treatments such as surgical excision or other effective local therapies [12].

High-risk BCCs, on the other hand, show characteristics that make them more difficult to treat. They can be larger tumors, located in critical anatomical areas (such as near the eyes, nose, or ears), or exhibit features that increase the risk of recurrence or involvement of larger areas of skin. They have a higher tendency for positive surgical margins and recurrence, particularly if not treated with sufficient rigor. Due to their invasive nature, early detection and treatment are crucial to prevent local tissue destruction [13].

## 3. Pathological Features of BCC

### 3.1. Generalities

BCC is a family of carcinomas arising from epithelial cells of either the epidermis or hair follicle stem cells [14], and it is made up of atypical basaloid elements, with hyperchromatic nuclei and scant cytoplasm, arranged in variably sized nests and strands, resulting in a “blue” basophilic appearance of the tumor in H&E-stained sections. Neoplastic aggregates usually show peripheral palisade, cleft artifacts and stromal fibromyxoid changes. Other common features are brisk mitotic activity, apoptotic cells, necrosis, calcification, keratin-derived amyloid deposition, superficial ulceration and pigmentation, the latter due to the presence of colonizing melanocytes and/or dermal melanophages [14,15]. No precursor lesions are recognized, but a background of solar elastosis is almost constantly seen [16]. Regression can be observed [17].

According to NCCN guidelines, BCC can be categorized in two pathological risk groups according to their morphologic features (*vide supra*): low-risk (comprising nodular, superficial, infundibulo-cystic and fibroepithelial histotypes) and high-risk (comprising infiltrating, basosquamous and sarcomatoid histotypes); furthermore, perineural invasion is considered a pathological hallmark of high-risk BCC. A rough correlation between the macroscopic appearance and the histological pattern exists. Moreover, it is important to note that different histotypes can be found within the same tumor; regardless, the high-risk component prevails in the attribution to the risk class [14].

### 3.2. Immunophenotype

**Figure 1:**
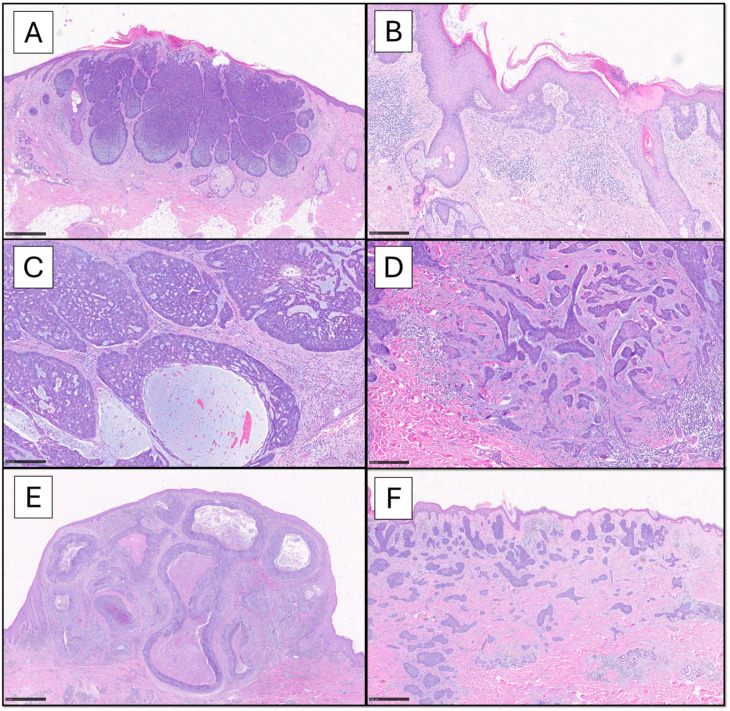
The panel shows the BCC histotypes mostly encountered in common practice. The nodular pattern is characterized by circumscribed proliferation of large nests, with a variable architecture ranging from solid ((**A**), scale bar 500 µm) to adenoid-cystic ((**C**), scale bar 250 µm) to frankly cystic (nodulocystic, (**E**), scale bar 1 mm); myxoid stromal changes are observed, especially within the nests (**C**). The superficial pattern ((**B**), scale bar 250 µm) is characterized by buds of neoplastic cells emanating from the basal layer of the epidermis and confined to papillary dermis. On the other hand, infiltrative pattern ((**D**), scale bar 250 µm) shows irregular strands of atypical basaloid cells, with spiking projections, embedded in a fibromyxoid and desmoplastic stroma. The micronodular variant ((**F**), scale bar 500 µm) is predominantly made up by small nests diffusely extending into the dermis, in the absence of defined boundaries (Hematoxylin-Eosin 4x-20x).

BCC is characterized by the expression of high-molecular-weight cytokeratins (HMWCKs) like CK-AE1/AE3, CK-5/6, CK-14, BerEP4/EpCAM (a membrane glycoprotein absent in normal squamous epithelium or SCC), BCL2, p63, CD10 (positive in tumor cells and negative in stroma), androgen receptor (AR) and p53. In contrast to adnexal-derived tumors, BCC usually does not stain with antibodies against CK-20, due to the absence of colonizing Merkel cells. CK-7, carcino-embryonic antigen (CEA) and epithelial membrane antigen (EMA) are usually negative [14,18]. Moreover, actin and neuroendocrine marker expression can be observed in more aggressive subtypes [17,18]. Figure 1 summarizes the histological variants of BCC.

### 3.3. Low-Risk BCC

**Nodular BCC** is the most common encountered histotype. It typically presents as a pearly or translucent papule or nodule with raised, rolled borders and often a central ulceration. The lesion is usually well-defined and has a smooth surface with telangiectasias (i.e., small blood vessels) visible on the surface [6,19,20] and is generally painless, but can bleed or crust especially if ulcerated [20]. It is mostly located on the face, particularly around the nose, eyelids, and ears, though it can also appear on the neck or chest [1,20]. It is characterized by large, well-circumscribed neoplastic nests centered in the dermis with or without epidermal or follicular connection [14,15]. The nests can be heterogeneous in dimension and architecture, showing peripheral palisading with cleft artifacts and central haphazard arrangement. The surrounding stroma is sparsely cellular but typically lacks desmoplasia. Numerous variants are known: keratinizing BCC, exhibiting central areas of mature keratinization within the nests; cystic/nodulocystic BCC, characterized by central cystic degeneration; adenoid-cystic BCC, with reticulate growth in a prominent mucinous stroma, resulting in mucinous pseudocyst formation and cribriform appearance. Further and less common histological patterns include BCC with adnexal differentiation (eccrine, apocrine, matrical, sebaceous, rippled/labyrinthine), as well as BCC with clear cell, signet-ring, granular and giant cell/pleomorphic features [21,22].

**Superficial BCC** presents as a flat or slightly elevated patch, often pink or red in color, with well-defined borders. The surface may appear shiny or scaly, and the lesion tends to be more erythematous and less raised than the nodular form. It is mostly found on the trunk, shoulders or upper arms, although it can also appear on the face. These lesions are typically asymptomatic, although they can occasionally itch or cause mild irritation [1,2,4,20]. Histologically, it comprises lobules of neoplastic cells with peripheral palisading associated with myxoid stroma, retraction artifact and lichenoid band-like inflammatory infiltrate, which are confined to the papillary dermis with extensive connection to the overlying epidermis or occasionally to the adnexal epithelium [14,15]. Clear cell changes can also be noted [21].

**Infundibulocystic BCC**, a peculiar and independent variant of BCC with adnexal differentiation towards the follicular infundibulum, presents as a well-circumscribed, symmetric nodular tumor, often located on the face or scalp [23]. These lesions may macroscopically resemble cysts or sebaceous lesions, with a firm, sometimes cystic feel [24]. It consists of anastomosing cords and nests of neoplastic cells, interspersed with small, infundibular cyst-like structures. The overall picture is of a symmetric well-delineated nodular tumor [14,15].

**Fibroepithelial BCC** (Pinkus Tumor) consists of a flat, pinkish or flesh-colored, soft nodular lesion resembling a fibroma or an intradermal nevus, often appearing as a small, indurated plaque with well-defined borders [25]. This variant is most commonly found on the trunk or scalp, tends to be asymptomatic and is often mistaken for other benign skin lesions [25,26]. It is defined by a circumscribed proliferation of thin, inter-anastomosing strands of basaloid cells emanating from the epidermis and surrounded by abundant loose fibrous stroma, resulting in a characteristic fenestrated appearance, with adjacent conventional BCC areas that can be found [14,15,27]. Some authors consider this tumor a variant of trichoblastoma, due to the frequent presence of stromal cell condensation around the neoplastic strands (reminiscent of hair germinative differentiation), the low proliferative activity and the possible colonization by CK20+ Merkel cells [14,15,26].

### 3.4. High-Risk BCC

**Infiltrating BCC** usually presents as an ill-defined, irregular, and often ulcerated lesion [28]. It is commonly found in conjunction with preexisting BCCs of other subtypes, particularly the nodular type. The lesion may appear as a plaque or a mass with indistinct borders, may often bleed easily and may show signs of deep tissue invasion, such as the destruction of surrounding structures [28]. Sometimes they can resemble a scar or a plaque reminiscent of morphea (morpheaform BCC) [29,30]. It typically develops in areas like the face, particularly around the eyes, nose, and ears. Histologically, it is characterized by diffuse infiltrative growth of irregular narrow, jagged strands of neoplastic cells, with poorly circumscribed boundaries and spiking projections, while peripheral palisading and cleft artifacts are usually focal. Perineural invasion can be observed, while lymphovascular invasion is exceptional [14,15,31]. To date, this category encompasses also sclerosing/morphoeic and micronodular BCCs, thus enhancing diagnostic concordance in identifying high-risk cases. The morpheic/sclerosing variant consists in an infiltrative BCC with very narrow strands of neoplastic cells embedded in a prominent desmoplastic stroma, deeply extending through the subcutaneous tissue. The micronodular variant is made up predominantly (i.e., >50% of tumor volume) by small nests of neoplastic cells showing diffuse, infiltrative, tentacular growth into the dermis, resulting in frequent invasion of subcutaneous tissue and extension to the margins [14,15].

**Basosquamous BCC**, formerly known as metatypical BCC, is a mixed type that combines elements of both BCC and squamous cell carcinoma (SCC). It typically presents as an ulcerated, firm nodule or plaque, with areas of keratinization and an irregular, raised edge. The lesion may resemble a poorly defined BCC or a more aggressive SCC. It often appears on the face, is prone to rapid growth, and shows a higher risk of recurrence and metastasis than classic BCC [32]. Histologically, it shows distinct zones of BCC (usually infiltrative type) intermixed with SCC, in varying proportions. There may be an area of transition between the two growth patterns, or they may be closely intertwined. BerEP4 is useful in demonstrating the two distinct zones, and it is important not to confuse this entity with keratinizing BCC, which we mentioned above [14,15].

**BCC with sarcomatoid differentiation** is an extremely rare and highly aggressive subtype of BCC. It presents as a large, firm, ulcerated mass with irregular borders, macroscopically resembling a malignant sarcoma [33,34]. These lesions tend to grow rapidly and can be painful or cause significant tissue damage. Recurrence rates are high, and these tumors can cause substantial local destruction if not promptly addressed [34]. Histologically, it is made up of two different components, intermingled together: a usual epithelial BCC and a malignant stromal part that can differentiate towards a variety of histologies reminiscent of pleomorphic undifferentiated sarcoma (UPS), osteosarcoma, chondrosarcoma, leiomyosarcoma, rhabdomyosarcoma or myoepithelial carcinoma. The latter can show focal or absent staining for CKs or p63 [14,15,32,33,34,35].

### 3.5. Differential Diagnosis

Histologic mimics of BCC may include nonneoplastic processes (follicular induction over dermatofibroma, basaloid follicular hamartoma), benign adnexal tumors (especially follicular-derived ones such as trichoblastoma/trichoepithelioma) or cutaneous carcinomas with basaloid or blue-cell features (sebaceous carcinoma (SB), Merkel cell carcinoma (MCC)). Discerning these entities requires clinical correlation, identification of key morphologic features and possibly ancillary tests including immunohistochemistry [18,36]. In particular, useful elements in histologic distinction with benign mimics are well circumscription, bland cytology (i.e., lack of atypia, significant mitotic activity or apoptosis/necrosis) and absence of stromal mucin or prominent clefting as well as evidence of true follicular differentiation (i.e., germinative buds and papillary mesenchymal bodies) and colonization by CK-20+ Merkel cells. SB and MCC show instead high-grade atypia, peculiar cytologic features (extensive sebaceous differentiation in SB, salt-and-pepper chromatin and nuclear molding in MCC), absence of peripheral palisading or clefting, possible intraepidermal pagetoid spreading and different immunophenotypical expressions [18,36].

## 4. Principles of Surgical and Topical Treatment

**Surgical excision** of BCCs is the primary therapeutic approach, consisting in the complete removal of the tumor with a rime of healthy tissue, in order to avoid microscopic positive margins and minimize the risk of recurrence [1,2,3,4,5,6,7,8,9,10,11,12,13]. The standard excisional free margin is typically 4 mm, almost when tissue preservation is not a primary concern, as this width successfully removes tumors less than 2 cm in over 95% of cases [37,38]. A recent systematic review suggested that a 3 mm margin could also be just as effective [39]. When the tumor is located on cosmetically sensitive areas, such as the face, Mohs micrographic surgery (MMS) may be employed, as it allows for precise removal of cancerous tissue while preserving as much healthy skin as possible [39,40,41,42]. The choice of surgical method depends on factors such as tumor size, location, and patient’s general conditions. Overall, surgical treatments correlate with the lowest recurrence rates, with MMS potentially resulting in better outcomes especially in high-risk BCC [40,43].

Topical therapies offer effective management options for low-risk BCC, particularly for specific body sites or patients not suitable for surgery. Overall, cure rates are approximately 10% lower than for surgical treatment modalities, but their recurrence rates are still acceptable, and cosmetic outcomes are likely better [43,44]. Imiquimod, an immunomodulator, stimulates the immune system to target and destroy tumor cells. It is approved for primary superficial BCC with a maximum tumor diameter of 2 cm [45]. **5-Fluorouracil (5-FU)** cream 5%, a pyrimidine antimetabolite causing DNA synthesis blockade, can also be used as an alternative to imiquimod; it is administered twice daily for 3–6 weeks [46]. Cryotherapy, which involves freezing the tumor with liquid nitrogen, is another treatment option for non-melanoma skin cancers, including BCC. It causes cytotoxic damage to the tumor by destroying blood vessels and inducing ice crystal formation within the cells. Cryotherapy is typically used for small, low-risk BCCs, but its major drawback is the inability to perform histological evaluation due to treatment-related tissue damage [47]. Photodynamic therapy (PDT) is another alternative, utilizing photosensitizing agents like methyl aminolevulinate (MAL), followed by activation with light. PDT is particularly useful for treating superficial BCC, especially when surgery is not feasible due to patient factors such as age or comorbidities. Although it has shown favorable results, recurrence rates and side effects, including erythema and swelling, may vary [48].

**Radiation therapy** (RT) is also sometimes to be considered as definitive therapy or in adjuvant, post-surgery settings [49]. Primary radiation therapy for non-surgical candidates can be extended to both low-risk and high-risk BCC, as well as to patients with advanced BCC [50]. Adjuvant RT can be considered an additional treatment option after multidisciplinary consultation for BCC with positive margins or in cases of extensive perineural invasion and/or large-nerve involvement [51]. A recent systematic review and meta-analysis for primary BCC comparing 5 nonrandomized studies and 40 randomized controlled trials with variable follow-ups reported recurrence rates of 3.5% after radiotherapy and 3.8% after Mohs surgery [52].

Despite their low metastatic potential, BCCs require regular monitoring due to their tendency to recur, especially in high-risk cases. Long-term follow-up is essential to detect any regrowth or new lesions, particularly in patients with a history of multiple BCCs.

## 5. Implications of Oncological Treatments

Systemic treatment is mandatory in mBCC, a rare challenging condition. Historically, available options were limited, but recent advances in targeted therapies, immunotherapy, and radiotherapy have expanded the landscape. To date, HHIs, such as vismodegib and sonidegib, represent the cornerstone of mBCC treatment. However, resistance to HHIs has highlighted the need for alternative therapies, including immunotherapy and chemotherapy [49].

### 5.1. Targeted Therapy: Hedgehog Pathway Inhibitors

The Hedgehog signaling pathway plays a pivotal role in the pathogenesis of BCC. Approximately 90% of BCCs demonstrate aberrant activation of this pathway, due to mutations in the *PTCH1* or *SMO* genes. This discovery led to the development of HHIs, which revolutionized the treatment of laBCC and mBCC [53,54].

In this field, **Vismodegib** was the first HHI to be approved for mBCC. The ERIVANCE BCC trial demonstrated that vismodegib led to an objective response rate (ORR) of 48% in mBCC patients, with a median progression-free survival (PFS) of 9.5 months and overall survival (OS) of approximately 33 months. Its approval marked a pivotal moment in the treatment of mBCC. The analyzed histological subtypes were infiltrative, micronodular, nodular and superficial BCC; nodular subtype has the better ORR, while the infiltrative subtype has the worse one [54].

**Sonidegib**, an oral inhibitor of SMO receptor, is approved for the treatment of laBCC and mBCC unsuitable for surgery or RT. In clinical studies, sonidegib has demonstrated substantial efficacy, particularly in patients with laBCC. ORRs have been shown to be around 44–58%, depending on treatment duration and population characteristics [55]. However, efficacy in mBCC tends to be lower, reflecting the more aggressive nature of this condition. In the BOLT trial, sonidegib demonstrated an ORR of 43% with a median PFS of 13.1 months in mBCC patients [56]. Histological subtypes that seem to respond more favorably to sonidegib include nodular and superficial BCC, which are more commonly associated with less aggressive clinical behavior. In contrast, more aggressive subtypes such as basosquamous carcinoma or morpheaform may show reduced sensitivity to this drug, possibly due to divergent molecular pathways involved in their pathogenesis or presence of higher stromal component, which can influence drug penetration and efficacy [55,56].

While HHIs have improved patient outcomes, their use is often limited by side effects such as muscle cramps, dysgeusia, and alopecia, that plays a critical role in patient adherence and long-term outcomes, especially in challenge scenarios where cumulative toxicity might influence retreatment decisions [49,55]. Additionally, resistance to HHIs remains a challenge, with some tumors developing mutations that bypass Hedgehog pathway inhibition [49].

### 5.2. Immunotherapy

Immunotherapy can be utilized in advanced BCC when resistance to HHIs occurs. In particular, checkpoint inhibitors targeting PD-1/PD-L1 demonstrated potential efficacy, primarily because of the tumor’s immunogenicity and immune checkpoint protein expression [57]. The sensitivity of BCC to immunotherapy correlates with multiple factors and seems to be primarily influenced by the expression levels of PD-1 and the number of tumor-infiltrating lymphocytes. In particular, some studies have shown that BCCs with higher PD-1 expression and increased tumor-infiltrating lymphocytes are more likely to respond to PD-1 inhibitors like pembrolizumab [58].

**Cemiplimab**, a PD-1 inhibitor, demonstrated efficacy in patients with HHI-resistant advanced BCC in the EMPOWER-BCC 1 trial [59], with an ORR of approximately 31%. While data on rare histological subtypes are limited, as mentioned above, their potentially higher mutational burden compared to common variants could render them more immunogenic and susceptible to immune checkpoint blockade. In summary, while the histological subtype of BCC provides valuable information about the tumor’s aggressiveness and potential behavior, the molecular and immunological characteristics predominantly influence the tumor’s sensitivity to immunotherapy. Therefore, assessing factors such as PD-1 expression and the presence of tumor-infiltrating lymphocytes can be helpful when considering immunotherapeutic approaches for BCC [59]. However, further investigations are needed.

### 5.3. Chemotherapy

Chemotherapy plays a limited but occasionally necessary role in the treatment of advanced BCC refractory to HHIs and immunotherapy. Given the rarity of mBCC, data on chemotherapy regimens are derived primarily from small case series and retrospective studies rather than large-scale randomized trials. The most commonly employed agents include platinum-based drugs, antimetabolites and taxanes, either as monotherapy or in combination.

**Platinum-based chemotherapy**, particularly cisplatin and carboplatin, has been used for mBCC due to its mechanism of inducing DNA crosslinking, leading to tumor cell apoptosis. Several studies have reported varying degrees of efficacy [60]. In a case series reported by Khandekar et al., two patients with mBCC treated with cisplatin monotherapy (100 mg/m^2^ IV every 21 days) achieved a complete response lasting 8 and 12 months, respectively [61]. Another retrospective study by Guthrie et al. evaluated cisplatin (50 mg/m^2^ IV on days 1 and 8) plus cyclophosphamide (600 mg/m^2^ IV on day 1) and doxorubicin (50 mg/m^2^ IV on day 1) every 21 days in seven patients with advanced BCC. The study reported a partial response rate of 43%, with a mPFS of 6 months [62].

**Taxanes**, particularly paclitaxel and docetaxel, have been used in combination regimens to enhance response rates. Jefford et al. reported the efficacy of treatment with carboplatin and paclitaxel every 21 days in an mBCC patient with a partial response after three cycles [63]. Docetaxel 35 mg/m^2^ and cisplatin 25 mg/m^2^ D1, D8 and D15, with one- or two-week intervals, were evaluated in a cohort of three patients with advanced BCC, who achieved CR [64].

In summary, while chemotherapy remains a non-first-line option for mBCC, it can provide clinical benefits in patients who have progressed on HHIs and immunotherapy. Further studies are needed to refine treatment strategies and identify biomarkers predicting chemotherapy response in mBCC.

### 5.4. Novel Therapeutic Approaches and Vaccines

Among the therapeutic innovations to be explored, further lines of immunotherapy as well as combination therapies involving immunotherapy and targeted therapy have been proposed.

**Pembrolizumab** was assessed in a small proof-of-concept study [65], where 16 participants received either pembrolizumab alone (9 patients) or in combination with vismodegib (7 patients). The ORR was 44% in the monotherapy group (four patients) and 29% in the combination group (two patients). Similarly, nivolumab as monotherapy was studied in 33 patients with advanced BCC following multiple lines of treatment in a phase II basket trial [66]. All participants had previously received HHI. Complete response, partial response, and stable disease rates were 12.5%, 18.8%, and 43.8%, respectively. Interestingly, a higher incidence of diabetes was observed, but no thyroid dysfunction, differing from adverse event profiles reported in metastatic melanoma treatment [66].

In addition, **nivolumab** was also evaluated in combination with ipilimumab or relatlimab in 24 patients with advanced BCC, including 5 cases of mBCC. Of these, 15 received nivolumab alone, 8 received nivolumab + relatlimab, and 1 received nivolumab + ipilimumab. The ORR for nivolumab monotherapy was 50% in the first-line setting and 20% in the second-line setting. Among the six evaluable patients in the nivolumab + relatlimab group, one experienced disease progression, four had stable disease, and one had a partial response. No responses were observed in the five patients with mBCC [67].

Primary and acquired resistance mechanisms to immunotherapy in BCC have been proposed. Walter et al. identified “cold” tumor characterized by the downregulation of MHC-I molecule expression and absence of infiltrating cytotoxic T-cells [68,69]. Sabbatino et al. described a lack of β2 microglobulin and HLA class I antigen expression in a patient resistant to frontline nivolumab [70]. Other studies have identified mechanisms such as altered interferon-gamma (IFNγ) signaling and increased expression of immune checkpoint proteins like LAG-3, which can mediate resistance [71,72]. Additionally, Dollinger et al. found macrophages over-represented in non-responders, although their role in resistance was unclear [72]. Probably, an immunosuppressive tumor microenvironment, with an abundance of immature dendritic cells and cytokines like TGF-β and IL-10, further contributes to resistance [73].

Numerous ongoing trials are exploring treatments for advanced BCC, including checkpoint inhibitors in neoadjuvant, adjuvant, and metastatic settings, summarized in Table 1.

Cemiplimab: Neoadjuvant use of cemiplimab in laBCC of the head and neck is being studied (NCT05929664). Combination therapy with cemiplimab and sonidegib is under evaluation in advanced BCC (NCT04679480). Another trial investigates cemiplimab with intratumoral Vidutolimod (TLR9 agonist) in solid tumors (NCT04916002).

Pembrolizumab: This is being studied in advanced resectable BCC of the head and neck in a neoadjuvant phase I trial (NCT04323202) and in combination with novel agents like MDNA11 (NCT05086692).

Nivolumab: A phase II trial is evaluating nivolumab as monotherapy or combined with relatlimab or ipilimumab in la- or mBCC (NCT03521830). Other trials are investigating combinations with talimogene laherparepvec for non-melanoma skin cancers.

Other Therapies: Adaptive dosing of vismodegib is under investigation (NCT05651828), and consolidative radiotherapy after vismodegib response is being studied in the RADIOSONIC trial (NCT05561634).

Novel intratumoral therapies include RP1 (oncolytic HSV-1 virus, ARTACUS trial) and KB707 (genetically modified HSV-1). Other emerging agents include CX-4945 (CK2 inhibitor) and oral PD-L1 inhibitors like INCB099318 (NCT04272034).

## 6. Conclusions

BCC remains the most prevalent skin cancer, with diverse clinical and pathological manifestations. While most cases are effectively managed with local treatments, rare and aggressive subtypes pose significant therapeutic challenges. The advent of HHIs has revolutionized the treatment of advanced BCC, yet resistance and adverse effects highlight the need for alternative strategies. Chemotherapy, though historically underutilized, may still play a role in select cases, particularly when other systemic therapies fail. Moreover, immunotherapy, particularly PD-1 inhibitors, represents a promising approach, though its efficacy varies based on tumor microenvironment factors rather than histological subtypes.

As the landscape of BCC treatment evolves, ongoing research into predictive biomarkers, novel targeted therapies, and combination strategies is crucial for optimizing patient outcomes. Future studies should also focus on refining treatment sequencing, improving patient selection for immunotherapy, and identifying possible mechanisms of resistance in order to overcome them.

## Figures and Tables

**Table 1:** Active clinical trials of new drugs in BCC.

Study ID	Phase	Drugs Investigated	Patients Enrolled	Endpoint	Status	Stage
NCT05929664	Phase II	Cemiplimab	Not specified	ORR and Disease Control Rate	Active	Locally advanced
NCT04679480	Phase II	Cemiplimab + Sonidegib	Not specified	ORR	Active	Advanced
NCT04916002	Phase II	Cemiplimab + Vidutolimod	Not specified	Efficacy in advanced solid tumors	Active	Advanced or metastatic
NCT04323202	Phase I	Pembrolizumab	Not specified	Safety and efficacy in neoadjuvant setting	Active	Resectable Advanced
NCT05086692	Phase I/II	Pembrolizumab + MDNA11	Not specified	Safety and efficacy	Active	Advanced
NCT05859074	Phase I	Pembrolizumab + MQ710	Not specified	Safety in dose escalation phase	Active	Not specified
NCT03521830 (ESMO 2023)	Phase II	Nivolumab ± Relatlimab ± Ipilimumab	Not specified	ORR	Active	Advanced or metastatic
NCT05651828	Phase II	Vismodegib (different schedules)	Not specified	Tolerability	Active	Advanced
NCT05561634 (RADIOSONIC)	Phase II	Radiotherapy after response to Vismodegib	Not specified	Safety and clinical benefit	Active	Locally advanced
ARTACUS Trial	Phase I/II	RP1 (oncolytic HSV-1)	Not specified	Safety and efficacy	Active	Advanced
NCT05970497	Phase I	KB707 (modified oncolytic HSV-1)	Not specified	Safety and tolerability	Active	Advanced or metastatic
NCT04272034	Phase I	INCB099318 (oral PD-L1 inhibitor)	32	Safety and tolerability	Active	Advanced
NCT05592626 (START-001)	Phase I/II	STAR0602 (TCR-targeting bispecific)	Not specified	Safety and efficacy	Active	Advanced
NCT05651828	Phase II	Adaptive therapy with Vismodegib	Not specified	Tolerability and safety	Active	Advanced
NCT05086692	Phase I/II	CX-4945	13	Safety and efficacy	Active	Advanced

## Data Availability

Not applicable.
